# Oligomerization of ZFYVE27 (Protrudin) Is Necessary to Promote Neurite Extension

**DOI:** 10.1371/journal.pone.0029584

**Published:** 2011-12-28

**Authors:** D. V. Krishna Pantakani, Marta M. Czyzewska, Anna Sikorska, Chiranjeevi Bodda, Ashraf U. Mannan

**Affiliations:** Institute of Human Genetics, University of Goettingen, Goettingen, Germany; Consejo Superior de Investigaciones Cientificas, Spain

## Abstract

ZFYVE27 (Protrudin) was originally identified as an interacting partner of spastin, which is most frequently mutated in hereditary spastic paraplegia. ZFYVE27 is a novel member of FYVE family, which is implicated in the formation of neurite extensions by promoting directional membrane trafficking in neurons. Now, through a yeast two-hybrid screen, we have identified that ZFYVE27 interacts with itself and the core interaction region resides within the third hydrophobic region (HR3) of the protein. We confirmed the ZFYVE27's self-interaction in the mammalian cells by co-immunoprecipitation and co-localization studies. To decipher the oligomeric nature of ZFYVE27, we performed sucrose gradient centrifugation and showed that ZFYVE27 oligomerizes into dimer/tetramer forms. Sub-cellular fractionation and Triton X-114 membrane phase separation analysis indicated that ZFYVE27 is a peripheral membrane protein. Furthermore, ZFYVE27 also binds to phosphatidylinositol 3-phosphate lipid moiety. Interestingly, cells expressing ZFYVE27^ΔHR3^ failed to produce protrusions instead caused swelling of cell soma. When ZFYVE27^ΔHR3^ was co-expressed with wild-type ZFYVE27 (ZFYVE27^WT^), it exerted a dominant negative effect on ZFYVE27^WT^ as the cells co-expressing both proteins were also unable to induce protrusions and showed cytoplasmic swelling. Altogether, it is evident that a functionally active form of oligomer is crucial for ZFYVE27 ability to promote neurite extensions.

## Introduction

The sprouting of neurite extension is a critical event in neuritogenesis, which serve as precursor for both axon and dendrites of the neuron. The budding and elongation of neurites in the developing neurons at the precise time and in the right direction is decisive for proper neuronal differentiation and connectivity [1]. Furthermore, in neurodegenerative disorders, changes in the pattern of neurite outgrowth have been observed. For the neurites to spike out from the cell soma or the parent axon; coordinated and widespread regulation of the cytoskeleton and membrane trafficking machinery are crucial [1], [2]. In terms of cytoskeleton remodeling, particularly, the long and stable microtubules (MT) of the parent axon must be locally severed into short highly mobile pieces that are able to shift into the newly forming branch sites to promote neurite outgrowth [3], [4].

In a recent study, it was shown that overexpression of spastin in neurons results in a dramatic increase in the neurite outgrowth (axonal branch formation) from the main axon [5]. Spastin is a MT severing enzyme, which belongs to AAA (ATPase Associated with various cellular Activities) family of proteins. Spastin shares high sequence homology with another well characterized MT severing protein P60-katanin in the AAA-region, however, the other regions of the proteins share little homology [5], [6]. Mutations in spastin are the most common cause for hereditary spastic paraplegia (HSP), a heterogeneous group of neurodegenerative disease affecting primarily the long axons of corticospinal tracts in the spinal cord [7], [8], [9], [10]. Interestingly, Yu and colleagues reported that depletion of spastin from neurons causes a significant reduction in the neurite formation, although it had minimal effect on the axon length [5]. In neurons, spastin has a far greater capacity to concentrate at the sites of branch formation and growth cones [5]. It has been shown that several spastin binding proteins are components of vesicular/membrane trafficking (atlastin, RTN1, CHMP1B, REEP1 and ZFYVE27) [11], [12], [13], [14], [15]. It is likely that spastin is locally recruited at the branch sites of the axon by its binding partner(s), where spastin may generate short MT, which then serves as precursor for neurite formation.

Remarkably, Shirane and Nakayama (2006) have shown that the spastin interactor ZFYVE27 (synonym protrudin) is also implicated in neurite formation by promoting directional membrane trafficking [16]. ZFYVE27 protein contains a Rab11 binding domain (RBD11) in its N-terminal region and FYVE domain in its C-terminal end. It also harbors a FFAT motif, a coiled-coil domain and spanned by three hydrophobic region (HR) motifs in the central region of the protein. These structural domains are hallmarks of a protein which might be implicated in membrane-cargo trafficking. Rab11 regulates membrane traffic at the trans-Golgi network–recycling endosome boundary and recycles them back to the plasma membrane [17], [18], [19]. Overexpression of ZFYVE27 in PC12 cell lines and primary hippocampal neurons lead to extensive neurite outgrowth [16]. Likewise, down-regulation of endogenous ZFYVE27 in PC12 cells by RNA interference results in inhibition of neurite outgrowth even after nerve growth factor induction and causes swelling of cell soma [16]. Overall, these data suggests that ZFYVE27 may play a vital role in neuronal development/differentiation.

In the database, ZFYVE27 was classified as a novel member of the FYVE family of protein, as its ∼70 residues FYVE domain preserves all the eight conserved cystine residues, which co-ordinate the binding of two zinc ions in a cross-braced topology [20], [21], [22]. The FYVE domain is suggested to be responsible for endosomal localization and majority of the FYVE finger proteins serve as regulators of endocytic membrane trafficking [23]. The targeting of FYVE proteins to the endosomal membrane is greatly influenced by the bi-/multivalent interactions of FYVE motif with PtdIns3*P* (phosphatidylinositol 3-phosphate) lipid moiety present in such membrane-compartment. The recruitment of FYVE protein to the endosomal membrane is enhanced by its oligomerization as this way multiple FYVE domains strengthen the interaction with PtdIns3*P* moiety in the membrane [24]. The most well characterized FYVE domain containing protein, EEA1 (early endosome antigen 1) has shown to form a parallel homo-dimer through its coiled-coil motif interaction [24], [25]. The homo-dimerization of EEA1 was shown to juxtapose two C-terminal FYVE domains, thus allowing simultaneous interactions with two PtdIns3*P* head groups [24]. Another FYVE protein, SARA was shown to intrinsically form a stable dimer and higher order oligomer through interaction with FYVE domain, which is detrimental for its endosomal localization [26], [27]. Moreover, a distinct anti-parallel homo-dimer has been reported for the FYVE domain of Hrs [28].

Based upon structural resemblance with other FYVE proteins, it can be postulated that ZFYVE27 might also form an oligomeric structure to render its function. In the present study, we demonstrate that ZFYVE27 is a peripheral membrane protein, which assembles into a homo-dimer/tetramer and the core interaction between the monomers is mediated primarily through the third hydrophobic region (HR3) motif of the protein. Moreover, we show that ZFYVE27^ΔHR3^ causes re-distribution of wild-type ZFYVE27 into the cytosol and deter its ability to promote protrusions in non-neuronal cells and neurites in neuronal cells.

## Results

### ZFYVE27 interacts with itself primarily through its HR3 region

**Figure 1:**
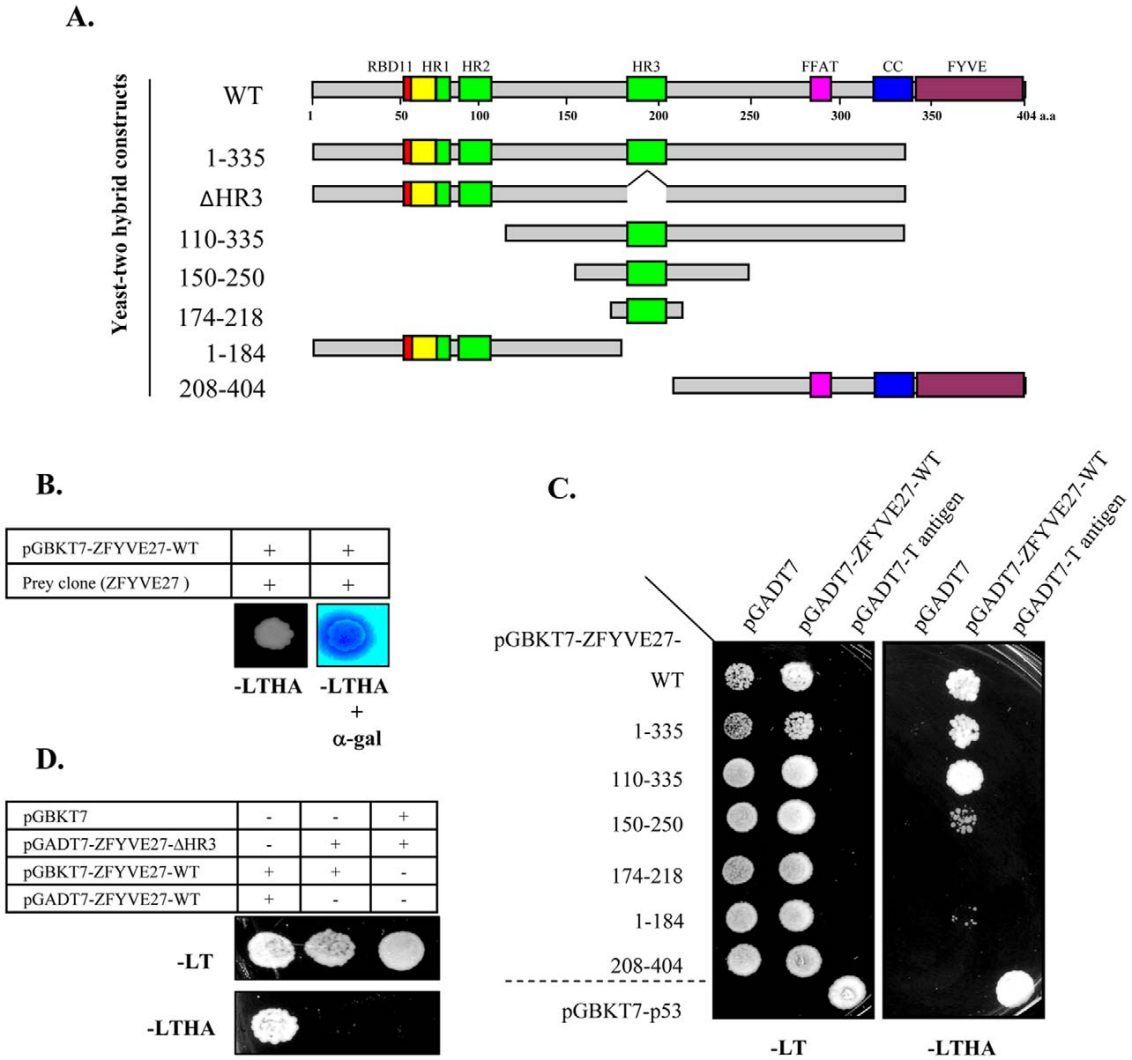
Yeast two-hybrid (Y2H) screen showing self-interaction of ZFYVE27. **(A)** Schematic diagram showing the structural domains of ZFYVE27 and delineation of various deletion constructs of ZFYVE27. ZFYVE27 contains Rab11 binding domain RBD11 (red) at its N-terminus and FYVE domain (purple) at C-terminus. The three hydrophobic regions (HR) are depicted in green and the overlapping region of HR1 region with the RBD11 motif is highlighted in yellow. The FFAT motif (pink) and the coiled-coil region (blue) mediate interaction with VAP-A protein. The generated deletion constructs for Y2H analysis are depicted thereof. **(B)** Activation of GAL4 reporter genes by interaction of ZFYVE27 with the prey clone (ZFYVE27) in the Y2H screen. A robust growth of yeast strain AH109 was observed on the nutritional selection medium -LTHA (lacking leucine, tryptophan, histidine and adenine) and also was positive for the α-galactosidase (α-gal) activity. **(C)** Determination of the core interaction region of ZFYVE27, which mediate self-interaction by direct-Y2H. The indicated deletion constructs of ZFYVE27 were fused with DNA binding domain of GAL4 and evaluated for their ability to interact with full-length ZFYVE27 fused to activation domain of GAL4 in Y2H experiments. The interaction between p53 and T-antigen was used as a positive control in the Y2H assay. **(D**) Evaluation of ZFYVE27-ΔHR3 (deletion of HR3 in ZFYVE27^1-335^ construct) interaction with full-length ZFYVE27.

We performed yeast two-hybrid (Y2H) screen on human fetal brain cDNA using full-length human ZFYVE27 as a bait protein in an attempt to identify ZFYVE27 interacting proteins. Through Y2H screen, we identified ZFYVE27 itself as its interacting partner (Fig. 1B). To verify the interaction of ZFYVE27 with itself and to delineate the domain of the protein which mediates this interaction, we generated several deletion constructs of ZFYVE27 for direct-Y2H assay (Fig. 1A). Direct-Y2H assay confirmed the interaction of full-length ZFYVE27 with itself (Fig. 1C). Furthermore, Y2H assay with generated deletion constructs revealed that the minimal protein fragment of ZFYVE27^150-250^ could interact with full-length ZFYVE27 (Fig. 1C). The ZFYVE27^150-250^ fragment harbors the HR3 (185-207 a.a) of the protein, when HR3 was deleted in ZFYVE27^1-335^, it failed to interact with full-length ZFYVE27 in the Y2H assay (Fig. 1D). The deletion construct ZFYVE27^1-184^ also showed a weak binding affinity towards the full-length ZFYVE27 (Fig. 1C).

### Interaction of ZFYVE27 monomers in the mammalian cells

**Figure 2:**
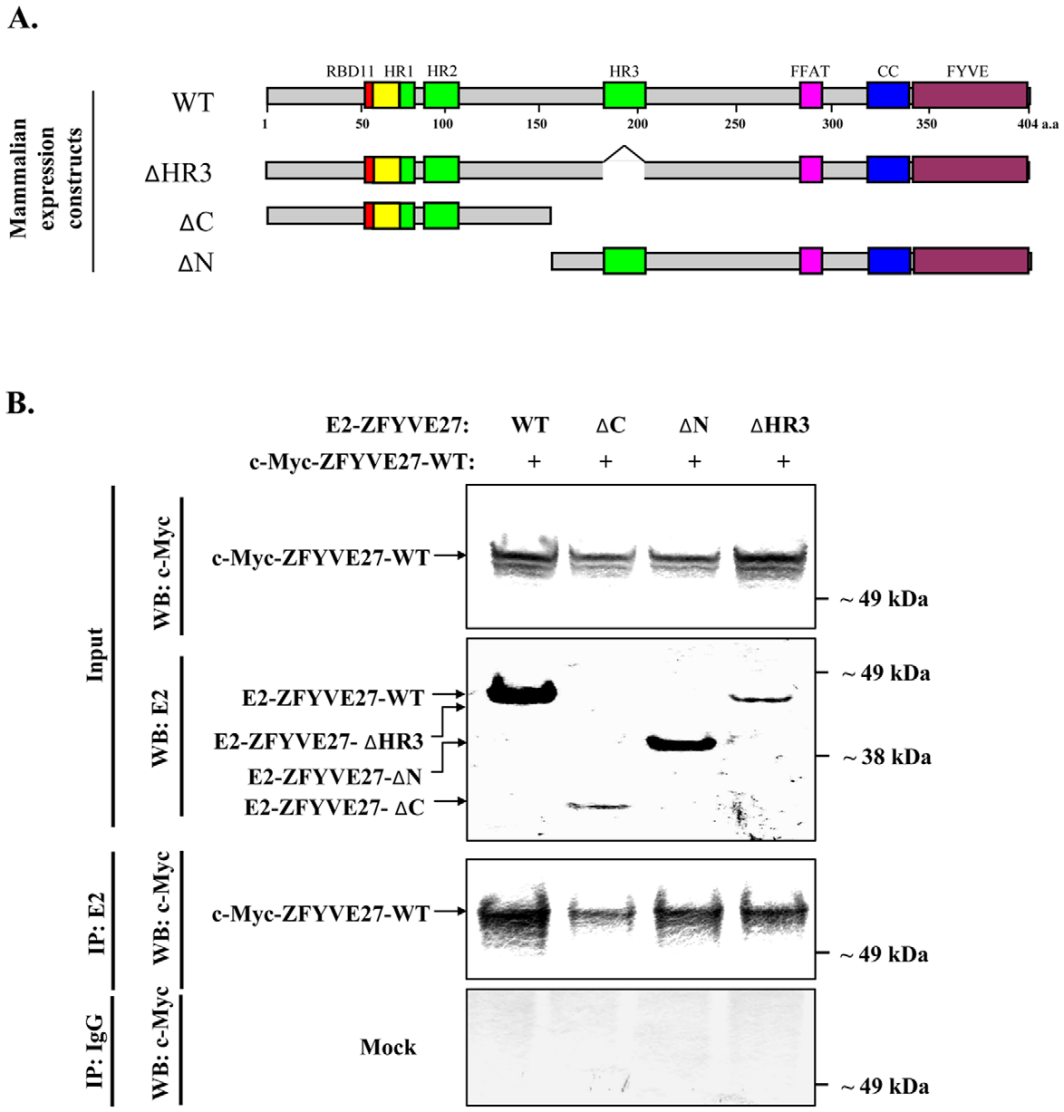
Co-immunoprecipitation assay to validate interaction of ZFYVE27 in mammalian cells. **(A)** Schematic representation of full-length and deletion constructs of ZFYVE27 for mammalian expression studies. **(B)** The full-length ZFYVE27 fused with c-Myc epitope tag was used for validation of its interaction with E2 tagged ZFYVE27, N-terminus (ΔC), C-terminus (ΔN) as well as with ZFYVE27 lacking the third hydrophobic region (ΔHR3) in NIH-3T3 cells. The cells were transiently transfected with respective constructs and subsequently co-immunoprecipitation was performed. The cell lysates were subjected to immunoprecipitation with E2 tag antibody and the resulting immunoprecipitants were analyzed in the immunoblot with c-Myc tag antibody (Co-IP: E2; WB: c-Myc). A portion of the cell lysates (input) was also subjected to immunoblot with either c-Myc (WB: c-Myc) or E2 (WB: E2) tag antibodies to verify the protein expression of the indicated constructs. For mock experiments, the cell lysates were precipitated with non-specific IgG and subsequently analyzed by immunoblotting (as described above).

To corroborate the ZFYVE27 self-interaction as observed in Y2H assay, also in mammalian cells, we performed co-immunoprecipitation experiments with transient co-transfection of full-length ZFYVE27 (ZFYVE27^WT^) tagged with c-Myc tag together with ZFYVE27^WT^ or with various deletion constructs tagged with E2 (Fig. 2A). Co-expression of E2-ZFYVE27^WT^ and c-Myc-ZFYVE27^WT^ in NIH-3T3 cell line and subsequent immunoprecipitation with E2-tag antibody and Western blot detection with c-Myc antibody revealed that E2-ZFYVE27^WT^, indeed, could interact with c-Myc-ZFYVE27^WT^ (Fig. 2B). Co-immunoprecipitation experiments with protein extract isolated from cells co-transfected with c-Myc-ZFYVE27^WT^ and one of the deletion construct (E2-ZFYVE27^ΔN(150-404aa)^ or E2-ZFYVE27^ΔC(1-150aa)^ or E2-ZFYVE27^ΔHR3(del 185-207aa)^) revealed self-interaction between the investigated proteins (Fig. 2B). We could confirm the interaction other way around by immunoprecipitation with c-Myc antibody and Western blot detection with E2-tag antibody (Fig. S1, supporting data). Since ZFYVE27 is known to interact with spastin [13], we analyzed the interaction between truncated ZFYVE27 and spastin. Co-expression of either E2-ZFYVE27^WT^ or E2-ZFYVE27^ΔHR3^ together with GFP-Spastin in NIH-3T3 cell line and subsequent co-immunoprecipitation revealed that spastin interact with wild-type as well as truncated ZFYVE27 (Fig. S2, supporting data).

**Figure 3:**
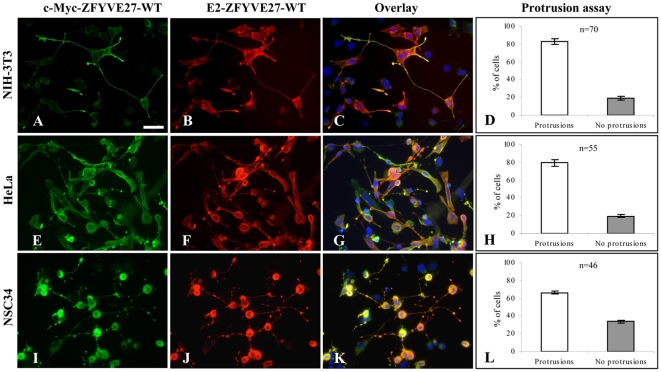
Co-localization of ZFYVE27 monomers in various cell lines. **(A–C)** The c-Myc-ZFYVE27^WT^ and E2-ZFYVE27^WT^ constructs were used to analyze the co-localization between ZFYVE27 monomers. Overexpression of both constructs in NIH-3T3 cell line revealed a pronounced formation of protrusions from cell soma and showed strong co-localization of ZFYVE27 monomers in the protruded structures. **(E–G)** A similar protrusions and co-localization was also observed in HeLa cells. **(I–K)** Overexpression of ZFYVE27^WT^ monomers in the NSC34 (a mouse embryonic spinal cord–neuroblastoma hybrid cell line) resulted in extensive neurite formation with bead-like structures. The ZFYVE27^WT^ monomers showed strongest co-localization in the neurite-beads. **(D, H, and L)** Protrusion assay → Quantification of proportion of the cells, doubly transfected (E2-ZFYVE27^WT^ and c-Myc-ZFYVE27^WT^) showing protrusions versus showing no protrusions. Scale bars – 50 µm (A–C, E–G and I–K); n, number of cells analyzed. Error bars indicate the standard deviation (SD) of protrusion assessment by two independent observers.

Furthermore, co-expression of E2-ZFYVE27^WT^ and c-Myc-ZFYVE27^WT^ in NIH-3T3 and HeLa cell lines confirmed the co-localization of both monomers, especially at the sites of protrusions (Fig. 3A-H). Similarly, overexpression of E2-ZFYVE27^WT^ and c-Myc-ZFYVE27^WT^ in the NSC34 neuronal cell line revealed that both monomers co-localize primarily at the neurite outgrowths as well as in the cell soma (Fig. 3I-L).

### ZFYVE27 assembles into a homo-dimer/tetramer

**Figure 4:**
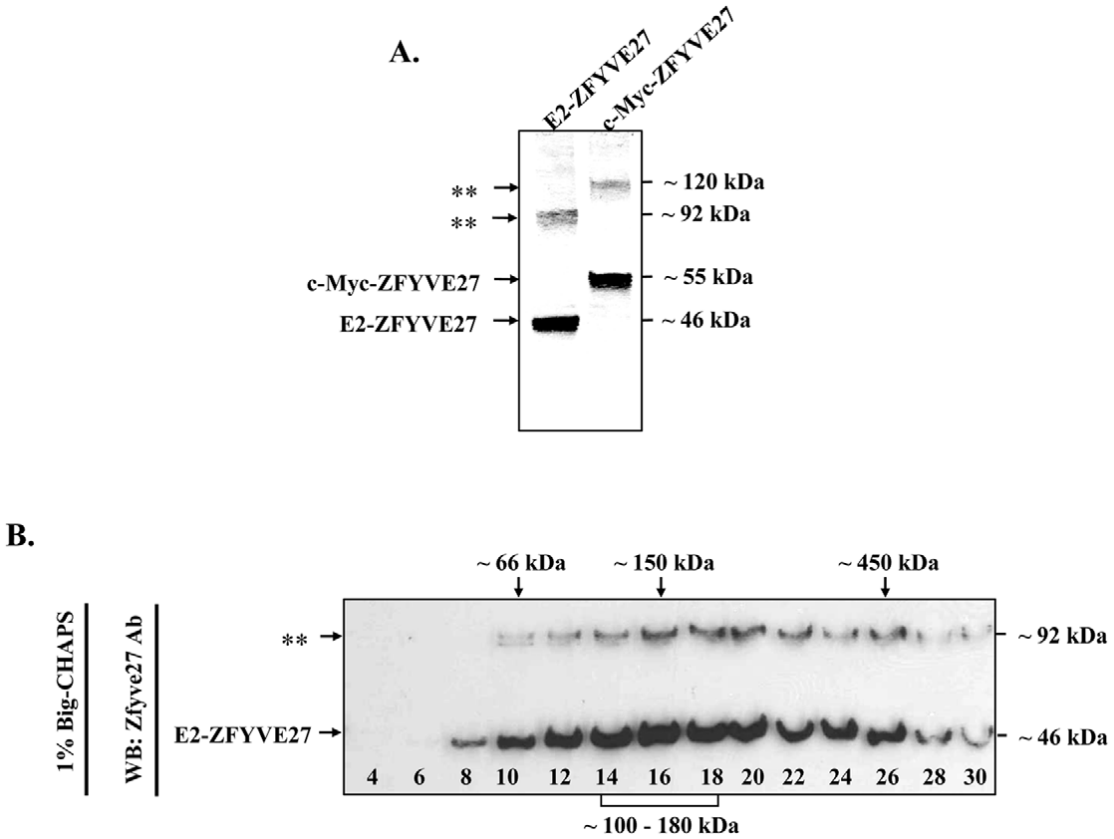
ZFYVE27 assembles into SDS-sensitive and –resistant dimer/tetramer. **(A)** The protein lysates from NIH-3T3 cells overexpressing either E2-ZFYV27^WT^ or c-Myc-ZFYVE27^WT^ were subjected to immunoblot with ZFYVE27 specific antibody. Asterisks denote the formation of SDS-resistant dimers corresponding to E2-ZFYV27^WT^ (∼ 92 kDa) and c-Myc-ZFYVE27^WT^ (∼ 120 kDa). Note: In pCS2-myc vector, six c-Myc epitope tags are fused in tandem therefore resultant c-Myc-ZFYVE27^WT^ protein is larger in size (∼55 kDa) as compared to E2-ZFYVE27^WT^ protein (∼46 kDa). **(B)** The NIH-3T3 cells transiently transfected with E2-ZFYV27^WT^ were solubilized in 1% Big-CHAPS and size-fractionated by 5-30% sucrose gradient centrifugation. The resultant fractions were analyzed by immunoblot with ZFYVE27 antibody; asterisks denote the formation of SDS-resistant dimers. The mobility of molecular weight markers in the sucrose gradient are indicated on the top.

In the immunoblots performed with protein lysates from the cells overexpressing ZFYVE27^WT^, we detected a distinct band, whose size corresponds to dimeric form of the protein in addition to the ZFYVE27 monomer (Fig. 4A). To validate the observed SDS-resistant dimeric form of ZFYVE27, we performed sucrose gradient centrifugation experiments with detergent solubilized cell lysates from NIH-3T3 cell line expressing E2-ZFYVE27^WT^. Towards this end, the membranes of the cells were solubilized with 1% Big-CHAPS and the native protein complex was isolated, which was then subjected to sucrose gradient centrifugation to fractionate the protein complex based upon their molecular size. The fractionated samples were resolved by SDS-PAGE and the oligomeric ZFYVE27 complex was visualized by immunoblotting with ZFYVE27 antibody. The immunoblot showed a diffused pattern with the migration of E2-ZFYVE27^WT^ with highest intensity in the fractions 14–18 corresponding to the molecular weight between ∼100–180 kDa indicating that the native ZFYVE27 complex exist as dimeric and/or tetrameric forms (Fig. 4B). In the immunoblot, the distinct SDS-resistant dimer also manifested a similar migration profile as the monomeric form of ZFYVE27 (Fig. 4B).

### The HR3 core interaction region of ZFYVE27 is essential for neurite extension

**Figure 5:**
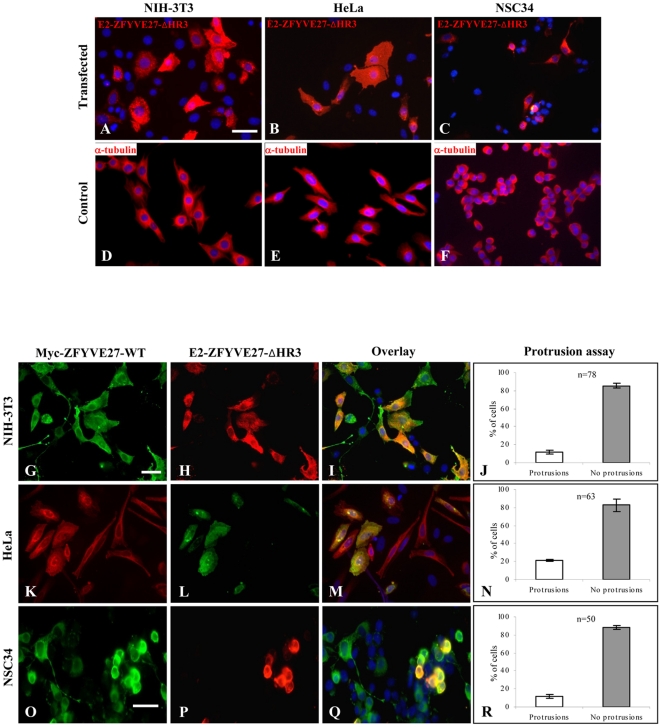
The truncated ZFYVE27^ΔHR3^ deters the ability of wild-type ZFYVE27 to promote directional membrane trafficking. **(A–C)** Overexpression of the mutant E2-ZFYVE27^ΔHR3^ (truncated ZFYVE27) failed to induce protrusions in all the analyzed cell lines (NIH-3T3, HeLa and NSC34) and caused cytoplasmic swelling with accumulation of protein either along endoplasmic reticulum or cytoskeleton. **(D–F)** Staining of control untransfected NIH-3T3, HeLa and NSC34 cells with α-tubulin showed no abnormal morphology. **(G–R)** Co-expression of both the c-Myc-ZFYVE27^WT^ and mutant E2-ZFYVE27^ΔHR3^ in various cell lines (NIH-3T3, HeLa and NSC34) showed the co-localization of both forms of ZFYVE27 in the cytosol. Notably, majority of cells co-expressing wild-type as well as ZFYVE27^ΔHR3^ failed to produce protrusions but rather caused swelling of the cytosol (I, M, and Q). Protrusion assay → Quantification of proportion of the cells, doubly transfected (E2-ZFYVE27^ΔHR3^ and c-Myc-ZFYVE27^WT^) showing cytoplasmic swelling (no protrusions) versus cells producing protrusions (J, N and R). Scale bars – 50 µm (A–C, D–F, G–I and K–M), 10 µm (O–Q); n, number of cells analyzed. Error bars indicate the standard deviation (SD) of protrusion assessment by two independent observers.

Since overexpression of ZFYVE27 induces protrusion/neurite formation in the cells [present study and [16], we sought to determine the functional relevance of the oligomeric ZFYVE27 in this process. Interestingly, overexpression of E2-ZFYVE27^ΔHR3^ failed to promote protrusions in all the analyzed cell types; rather, we observed accumulation of the ZFYVE27 protein in the cytosol, most likely either along the endoplasmic reticulum (ER) or cytoskeleton, leading to swelling of the cell soma (Fig. 5A–C). We did not observe these morphological changes in control untransfected cells, as expected (Fig. 5D–F). When we measured the width of the cytosol across the nucleus of cell, we detected statistically significant increase in soma size in ZFYVE27^ΔHR3^ expressing cells in comparison to ZFYVE27^WT^ expressing or control cells (Fig. S3, supporting data). Next, we co-expressed E2-ZFYVE27^ΔHR3^ together with c-Myc-ZFYVE27^WT^ to assess the effect of mutant protein on the wild-type ZFYVE27 intracellular distribution and function. Co-expression of the mutant E2-ZFYVE27^ΔHR3^ protein with c-Myc-ZFYVE27^WT^ inhibited the ability of wild-type protein to promote protrusions in both non-neuronal cell lines (NIH-3T3 and HeLa) (Fig. 5G–N) and neuronal (NSC34) cell line (Fig. 5O–R). Remarkably, mutant E2-ZFYVE27^ΔHR3^ caused redistribution of c-Myc-ZFYVE27^WT^ from the site of protrusions to the cytosol (Fig. 5I, 5M and 5Q).

**Figure 6:**
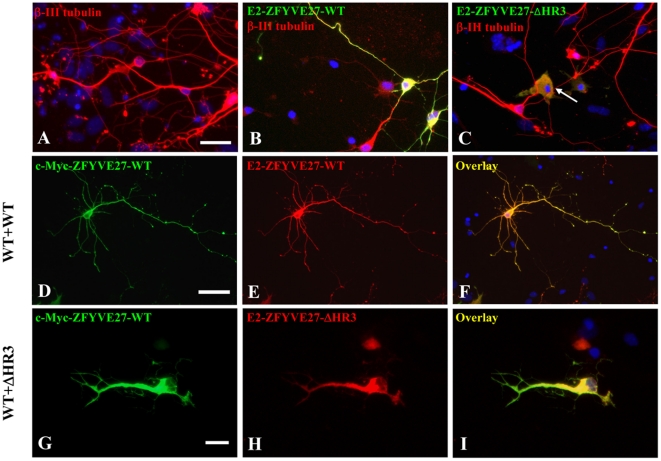
The truncated ZFYVE27^ΔHR3^ impairs the ability of wild-type ZFYVE27 to promote neurites in primary neuronal cells. (**A**) Control primary neurons stained for β-III tubulin, a neuronal marker. Overexpression of E2-ZFYVE27^WT^ (**B**) and E2-ZFYVE27^ΔHR3^ (**C**) in primary neuronal cells. Arrow in (**C**) showing the swelling of the cell soma of a neuronal cell expressing E2-ZFYVE27^ΔHR3^. (**D–F**) Overexpression of both wild-type ZFYVE27 (c-Myc-ZFYVE27^WT^ and E2-ZFYVE27^WT^) proteins revealed an enhanced neurites formation from the cell soma and showed co-localization of ZFYVE27 monomers. (G–I) Co-expression of both the c-Myc-ZFYVE27^WT^ and mutant E2-ZFYVE27^ΔHR3^ in primary neurons showed the co-localization of both forms of ZFYVE27 mostly in cell soma and the neuron failed to produce normal length axon as well as /dendrites. Scale bars – 20 µm (A–C), 50 µm (D–F), 10 µm (G–I).

Next, we evaluated the ability of ZFYVE27^WT^ and ZFYVE27^ΔHR3^ to promote neurite formation in neuronal cells by expressing them in primary neuronal culture. Neuronal cells expressing E2-ZFYVE27^WT^ showed a neuronal morphology similar to the control neurons (Fig. 6B). Interestingly, neuronal cells expressing E2-ZFYVE27^ΔHR3^ revealed swelling of the cell soma (Fig. 6C), similarly, as observed in both non-neuronal and neuronal cell lines (Fig. 5A–C). Further, co-expression of c-Myc-ZFYVE27^WT^ and E2-ZFYVE27^WT^ in primary neurons led to increased branch sites and enhanced neurite extension (Fig. 6D–F). In contrast, co-expression of both c-Myc-ZFYVE27^WT^ and truncated E2-ZFYVE27^ΔHR3^ in primary neuronal cells caused impairment of neuritogenesis (Fig. 6G-I).

### ZFYVE27 is a peripheral membrane protein

**Figure 7:**
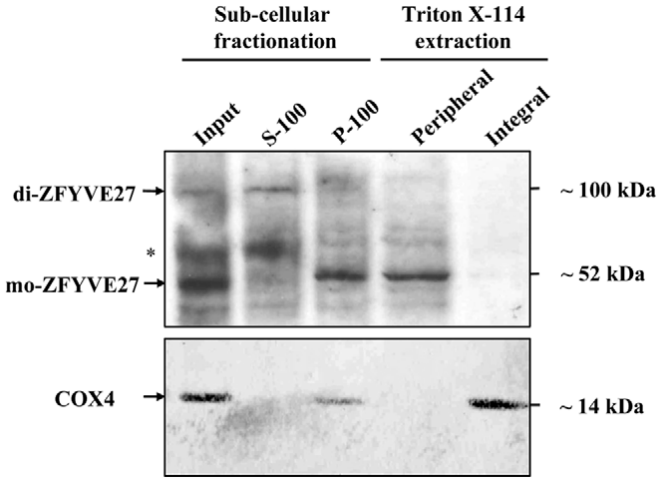
ZFYVE27 is a peripheral membrane protein. Sub-cellular fractionation was performed with NSC34 cell extract. The cytosolic fraction (S-100) and the membrane fraction (P-100) were analyzed by immunoblot with ZFYVE27 antibody. Asterisks denote the non-specific band. The monomeric ZFYVE27 (mo-ZFYVE27) and SDS-resistant dimeric ZFYVE27 (di-ZFYVE27) are indicated. The membrane fraction (P-100) was subjected to Triton X-114 phase separation and equal volumes of aqueous phase (peripheral) and detergent phase (integral) fractions were analyzed by immunoblotting. The blot was stripped and re-probed with COX4 antibody, a marker for the integral membrane protein.

To assess the membrane association properties of ZFYVE27, we used NSC34 cells expressing ZYFVE27 endogenously. Western blot analysis with ZFYVE27 antibody on protein isolated from membrane and soluble cytosol of NSC34 cells indicated that the monomeric ZFYVE27 localized into the membrane (Fig. 7). In contrast the SDS-resistant dimeric form of ZFYVE27 was enriched in the cytosolic fraction (Fig. 7). Next, the membrane fraction was treated with Triton X-114 to dissociate the peripheral membrane proteins from the membrane bilayer. Immunoblot analysis of the resultant aqueous and detergent fraction proteins after Triton X-114 membrane phase separation revealed that ZFYVE27 monomer is a peripheral membrane protein (Fig. 7).

### ZFYVE27 is a novel member of FYVE family, which binds to PtdIns3*P*

**Figure 8:**
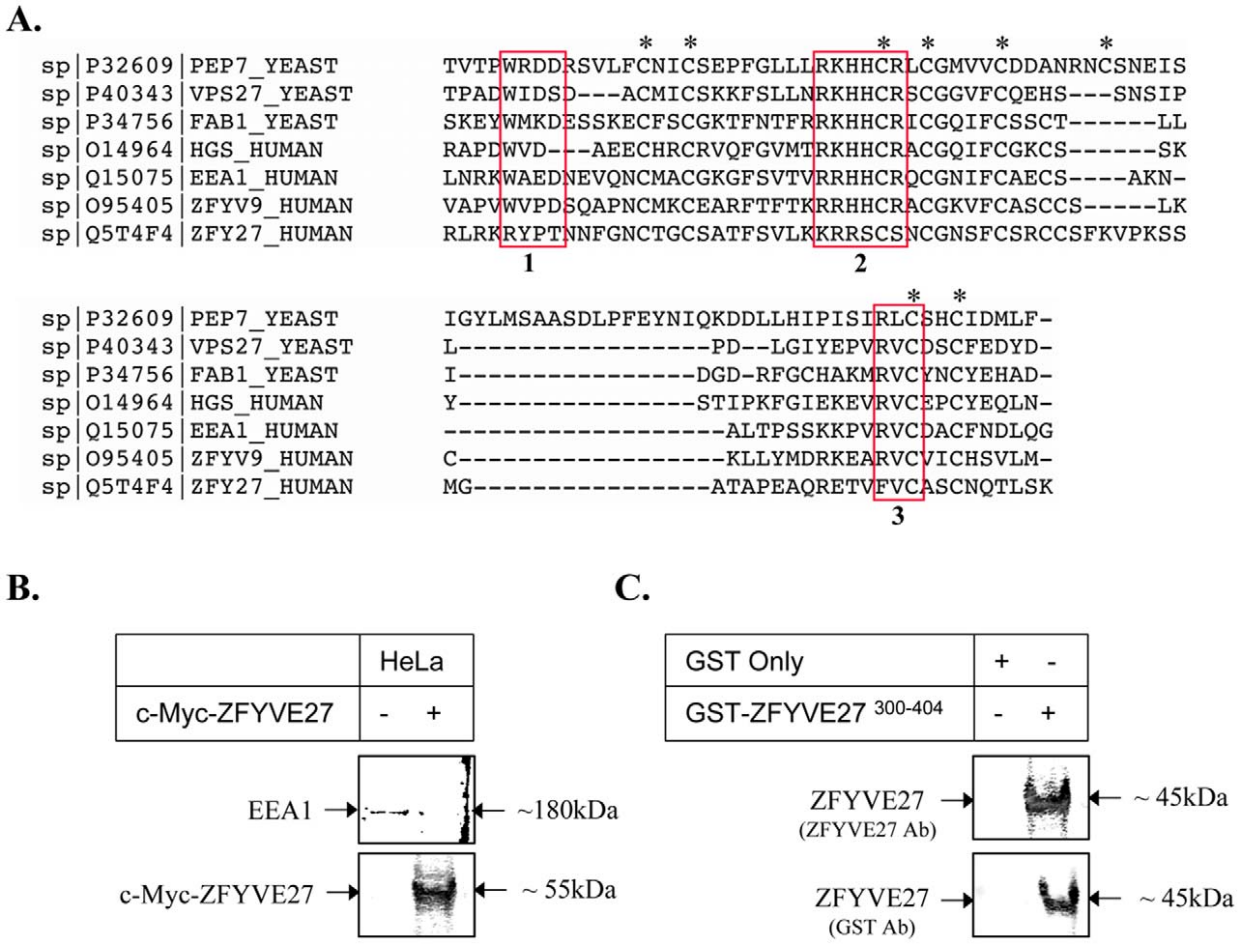
ZFYVE27 a novel member of FYVE family, which show binding affinity towards PtdIns3*P.* **(A)** Alignment of the FYVE domain of ZFYVE27 (ZFY27) with other FYVE family proteins; EEA1, ZFYVE9 (ZFYV9), HGS/HRS and yeast FAB1, PEP7, VPS27. The red boxes are highlighting the conserved WXXD motif (1), R(R/K)HHCR motif (2) and RVC motif (3). The conserved cysteine residues which co-ordinates the zinc ion binding were marked with asterisks. **(B)** The liposomal binding assay shows specific binding of c-Myc-ZFYVE27^WT^ to the PolyPIPosomes consisting of PtdIns3*P*. As a positive control, we also detected the binding of endogenous EEA1 to the PolyPIPosomes. **(C)** The recombinant GST-ZFYVE27^300-404^ protein also showed binding affinity to PtdIns3*P*. As a negative control, GST (alone) protein was used in the liposomal binding assay.

*In silico* analysis of the FYVE domain from ZFYVE27 revealed that although it has the conserved cysteine residues to co-ordinate the zinc ion binding, it lacks the conserved FYVE signature motifs; WXXD, RVC and R(R/K)HHCR (Fig. 8A), which facilitate the binding of FYVE domain to PtdIns3*P* specifically. To assess the PtdIns3*P* binding properties of ZFVYE27, we performed liposomal assay using PolyPIPosomes. Interestingly, our liposomal assay clearly showed that PtdIns3P containing PolyPIPosomes could pull down c-Myc-ZFYVE27^WT^ (Fig. 8B) and as a positive control; we could detect the binding of endogenous EEA1 to PolyPIPosomes (Fig. 8B). To rule out the possibility of indirect binding of ZFYVE27 to PtdIns3*P* via some linker proteins, we used recombinant ZFYVE27 (GST-ZFYVE27^300-404^) in the liposomal assay. The GST-ZFYVE27^300-404^ protein also showed binding specificity for the PtdIns3*P* (Fig. 8C).

## Discussion

ZFYVE27 was initially identified as a spastin binding protein [13]. Subsequently, Shirane and Nakayama (2006) has shown that ZFYVE27 (Protrudin) plays a crucial role in membrane trafficking in cells [16]. When ZFYVE27 was overexpressed in neuronal and non-neuronal cells, it induced neurites and protrusions, respectively, from the cell soma. ZFYVE27 along with Rab11 was shown to act as important determinant of the directional membrane trafficking and this activity is essential for neurite outgrowth in neuronal cells [16], [19]. To gain mechanistic insights into the role of ZFYVE27 in directional membrane transport during neurite formation, we attempted to identify proteins which interact with ZFYVE27 by means of Y2H screen.

Through Y2H assay, we identified ZFYVE27 as its own interaction partner, suggesting that ZFYVE27 form an oligomer. We confirmed the interaction of ZFYVE27 with itself by direct-Y2H assay. Next, to delineate the domain/motif through which ZFYVE27 self-associate, we generated a series of deletion constructs of ZFYVE27 and assessed their interaction with full-length ZFYVE27 by direct-Y2H analysis. By means of these deletion constructs, we could map the core interaction region to HR3 (185-207 a.a) motif of ZFYVE27. Next, we also showed that ZFYVE27 interact with itself in mammalian cells by co-immunoprecipitation and co-localization studies. The co-immunoprecipitation studies indicated that the protein fragments; ZFYVE27^ΔN^, ZFYVE27^ΔHR3^ and ZFYVE27^ΔC^ were also able to interact with full-length ZFYVE27^WT^. These data suggests that several regions of the ZFYVE27 protein serve as stabilizer for the oligomeric structure. In particular, the coiled-coil region in the C-terminus and HR1/HR2 motifs in the N-terminus could serve as stabilizer for ZFYVE27 oligomer. It was shown that the coiled-coil region of EEA1 is critical for homo-dimerization [24]. Surprisingly, when assessed by direct-Y2H assay, the deletion of HR3 (ZFYVE27^ΔHR3^) was sufficient to abolish its interaction with ZFYVE27^WT^. The discrepancy between co-immunoprecipitation as compared to Y2H assay could be due to differential sensitivity of detection by these experimental systems. As Y2H assay reveals direct interaction between two proteins, however, co-immunoprecipitation will also detect indirect interaction among proteins in an oligomeric complex.

To decipher the oligomeric nature of ZFYVE27, we performed sucrose gradient centrifugation and showed that ZFYVE27 oligomerizes into dimer/tetramer forms. Although, majority of FYVE proteins form a dimer, they can further form a higher order quaternary structure as reported for Hrs [29]. Hrs was shown to form a hexamer and the oligomeric form of Hrs comprises a trimer of dimers [29]. Also, a quaternary structure of EEA1 has been postulated, where two dimeric EEA1 juxtapose and elicit its function as a tetramer [24]. Similarly, ZFYVE27 dimers might constitute a tetrameric structure.

Notably, we detected a SDS-resistant dimeric form of ZFYVE27 by immunoblot analysis, both in the fractions of sucrose gradient centrifugation as well as when conventional SDS-PAGE analysis was performed. In sucrose gradient centrifugation, the sedimentation properties of SDS-resistant form of ZFYVE27 was similar to that of SDS soluble ZFYVE27. The observation of SDS-resistant form is not unique to ZFYVE27 as it was also reported for EEA1 [25]. Perhaps, a covalent bond formation between two monomers during homodimerization leads to the formation of this SDS-resistant FYVE dimeric form.

Majority of FYVE proteins are peripheral membrane proteins and they are targeted to membrane surface by specific binding to PtdIns3*P*, which are located in specific cargo vesicles derived from endosome [23], [30], [31], [32]. The membrane translocation is further facilitated by additional interaction of FYVE protein with other membrane proteins, for example; in case of EEA1, its interaction with Rab5 significantly increases the avidity of binding of EEA1 to membrane [31], [32], [33], [34]. *In silico* prediction of ZFYVE27 protein structure indicates that it consists of three hydrophobic regions (HR1-3). A recent study reported that ZFYVE27 interacts with VAP-A protein and postulated that ZFYVE27 may be an integral membrane protein [35]. In contrast, our sub-cellular fractionation and Triton X-114 lipid/aqueous phase separation analysis revealed that ZFYVE27 is a peripheral membrane protein, which conforms to the membrane association properties of other FYVE family proteins, which are also peripheral membrane proteins [23], [30], [31], [32].

**Figure 9:**
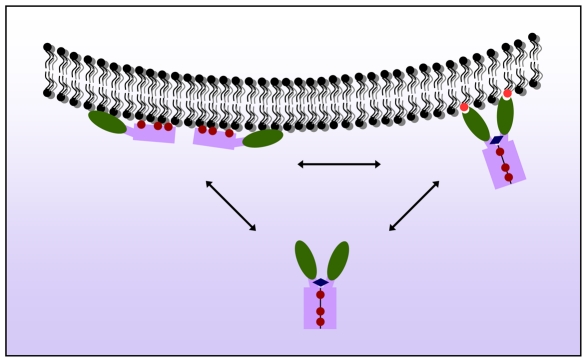
Schematic diagram illustrating a hypothetical model of ZFYVE27 dimerization. The monomeric form of ZFYVE27 can associate with the membrane through hydrophobic interactions with its three hydrophobic regions, namely: HR1-3 motifs (maroon filled circles), which might enable the monomers to embed as a peripheral membrane protein. In the cytoplasm, ZFYVE27 can exist as a soluble dimer, which could possibly be formed and stabilized by the self-interaction of three HRs and the coiled-coil domain (blue diamond) between two monomers. The dimerization of ZFYVE27 could most likely occur on the membrane.

Remarkably, the SDS-resistant dimeric form of ZFYVE27 was detectable exclusively in the soluble cytosolic fraction. It can be envisaged that in a monomeric form of ZFYVE27, the three HR regions are probably masked by its interaction with membrane (Fig. 9). The homodimerization of ZFYVE27 on the surface of the membrane might have been facilitated by the hydrophobic-hydrophobic interaction between the HR regions among the monomers. In the homodimeric form, the HR regions might reside in the core interface region, which is then probably shielded from the hydrophilic cytosol, thus, rendering a stable soluble dimeric ZFYVE27 in the cytoplasm (Fig. 9). The physiological function of a soluble ZFYVE27 dimer is not yet evident, but its ability to exist as a soluble form may allow itself to dock to various vesicular compartments swiftly, which could allow ZFYVE27 to promote directional membrane trafficking efficiently.

The FYVE proteins are targeted to endosomal membrane due to their ability to specifically bind to head-group of PtdIns3*P* lipids, which are localized in the endosomal vesicles [23]. The conserved FYVE signature motifs; WXXD, RVC and R(R/K)HHCR are critical for specific binding to PtdIns3P moiety [26], however *in silico* analysis revealed that ZFYVE27 show minimal sequence conservation at these signature motifs. *In vitro* liposome binding assay revealed that ZFYVE27 still retains the ability to bind to PtdIns3*P* moiety. Although, the FYVE domain of several proteins show binding specificity to PtdIns3*P in vitro*, but when expressed in cells, many FYVE proteins show a different subcellular localization than to endosomes [36], [37], therefore our finding should be interpreted with discretion. In this context, it is relevant to mention that in the neuronal cells, ZFYVE27 localizes in the ER under basal conditions; however, it translocates to recycling endosomes (RE) in response to nerve growth factor stimulation and through RE trafficked to the sites of neurite extension [16]. ZFYVE27 contain a FFAT motif, which could be responsible for its ER localization [35]. The FFAT motif is present in several lipid-binding proteins, which are involved in the transport of lipids between the ER and the Golgi apparatus [38], [39]. Additionally, the ER localization of ZFYVE27 could be aided by its interaction with VAP-A and Rab11, which are shown to localize to ER [16], [35]. Interestingly, the Rab11 is a core component of RE [17], [18], [22], it is likely that translocation of ZFYVE27 to RE is facilitated by its interaction with Rab11.

Overexpression of ZFYVE27 promotes neurite extension from the cell soma and ZFYVE27 is concentrated in the tip of the protruding neurite [16]. Surprisingly, overexpression of truncated ZFYVE27^ΔHR3^ in cells failed to induce protrusions/neurites rather caused swelling of cell soma and the mutant protein showed accumulation either along the ER or cytoskeleton. The cytoplasmic swelling could be due to the inability of ZFYVE27^ΔHR3^ to promote directional membrane trafficking thus leading to accumulation of membrane cargoes within the cytosol. To evaluate the effect of ZFYVE27^ΔHR3^ on the function of olgiomeric ZFYVE27, we co-expressed it with ZFYVE27WT in both neuronal and non-neuronal cell types; the ZFYVE27^ΔHR3^ exerted a dominant negative effect on ZFYVE27^WT^. The cells co-expressing ZFYVE27^WT^ and ZFYVE27^ΔHR3^ were also unable to produce protrusions and showed cytoplasmic swelling as observed for cell expressing only ZFYVE27^ΔHR3^. Moreover, the ZFYVE27^WT^ was re-distributed into the cytosol along with ZFYVE27^ΔHR3^. The co-immunoprecipitation studies revealed that the truncated ZFYVE27^ΔHR3^ still retains the ability to interact with ZFYVE27^WT^ to form oligomer. It was also able to bind to spastin. Although, a hetero-oligomeric complex of ZFYVE27^WT^/ZFYVE27^ΔHR3^ is non-functional as it could not promote protrusion/neurite extensions rather caused cytoplasmic swelling. Overall, our findings suggest that a functionally active form of oligomer is essential for ZFYVE27 ability to promote neurite extensions in neurons and highlights the role of HR3 motif in directional membrane trafficking.

Interaction of ZFYVE27 with spastin, VAP-A/B and KIF5A highlights its role in motor neuron disease [13], [35], [40]. Mutations in spastin and KIF5A are the primary cause for HSP [7], [8], [9], [10], [41] and mutation in VAP-B causes amyotrophic lateral sclerosis (ALS) [42], [43]. Both diseases share overlapping symptoms and mutation in spastin has been identified in the ALS patient [44]. It is interesting to note that overexpression of spastin in neurons causes a dramatic increase in the neurite extension formation [5]. It is probable that spastin, through it's interaction with ZFYVE27 could be recruited to the sites of neurite formations, where spastin can produce short MT by local severing and facilitate the promotion of the neurite extension [2], [3], [4]. Similarly, it was shown that interaction of ZFYVE27 with VAP-A is essential both for its localization to ER and for its ability to promote neurite extension [35]. Taken together, it is evident that ZFYVE27 along with the interaction partners such as spastin, VAP-A/B, Rab11 and KIF5A are components of a common cellular process, which may be essential for neuritogenesis. Dysfunction of this molecular pathway could be an underlying cause for the pathogenesis of motor neuron diseases such as HSP and ALS.

## Materials and Methods

### Yeast-two hybrid (Y2H) screen

We used the Matchmaker Two-Hybrid System kit (BD Clontech) for identification of putative ZFYVE27 interacting proteins. To generate the bait construct, the full-length human ZFYVE27 cDNA (GenBank accession No.: NM_001002262.2) was PCR amplified from human fetal brain cDNA and cloned into *EcoR* I and *BamH* I restriction sites of pGBKT7 vector (BD Clontech), resulting in a fusion of GAL4 DNA-binding domain with ZFYVE27. The pGBKT7-ZFYVE27 construct was transformed into AH109 yeast strain to test and exclude auto-activation of GAL4 activated reporter genes, *HIS3*, *ADE*, *LacZ*. The yeast two-hybrid screen was performed on a human fetal brain cDNA library (constructed in pGADT7-Rec plasmid) using a Matchmaker pre-transformed kit (BD Clontech). The matchmaker pre-transformed library in yeast strain Y187 was mixed and mated together with strain AH109 containing the ZFYVE27 bait construct. After 24 hrs of mating, the culture was spread on SD/-Leu/-Trp/-His/-Ade plates and the surviving colonies were verified on SD/-Leu/-Trp/-His/-Ade/+ X-α-Gal. The positive clones were cultured and the plasmid DNA was isolated using QIAprep Spin Miniprep Kit (Qiagen). The cDNA inserts of these plasmid clones were PCR amplified and sequenced. Identities of cDNA clones were determined by BLAST analysis (http://blast.ncbi.nlm.nih.gov/Blast.cgi).

### Direct Y2H assay

For mapping the core self-interaction region of ZFYVE27, several deletion bait (pGBKT7) and prey (pGADT7) constructs of ZFYVE27 were generated. Following fragments of ZFYVE27 were used for deletion constructs (as outlined in Fig. 1A); 1–335 a.a (amino acids) [corresponding nucleotides (nt) 201–1205 of NM_001002262.2], 110–335 a.a (nt 528–1205), 150–250 a.a (nt 648–939), 174–218 a.a (nt 720–854), 1–184 a.a (nt 201–752) and 208–404 a.a (nt 822–1412). For direct Y2H assay, the ZFYVE27 bait and prey constructs were co-transformed into AH109 strain by lithium acetate method [45]. The co-transformants were first selected on SD/-Leu/-Trp plates and later tested for the reporter gene expression on SD/-Leu/-Trp/-His/-Ade and X-α-Gal plates.

### Construction of mammalian expression plasmids

The pGBKT7-ZFYVE27 plasmid DNA was used as a template for the PCR amplification and cloning of ZFYVE27 into *BamH* I and *Kpn* I restriction sites of pQM-N-Tag A (Abcam) to generate E2-ZFYVE27^WT^ construct. To generate c-Myc-ZFYVE27^WT^ construct, ZFYVE27 ORF was cloned into *EcoR* I site of pCS2-myc expression vector [46]. [Note: In pCS2-myc vector, six c-Myc epitope tags are fused in tandem therefore resultant c-Myc-ZFYVE27 protein is larger in size as compared to E2-ZFYVE27 protein]. The E2-ZFYVE27 was used as a template for PCR amplification of a plasmid lacking HR3 (nt 750–822 of NM_001002262.2) using a modified QuickChange based PCR reaction (Stratagene) with primers complementary to the both 5′ and 3′ flanking regions of HR3 and generated the E2-ZFYVE27^ΔHR3^ construct. For the generation of E2-ZFYVE27^ΔC1-150^ and E2-ZFYVE27^ΔN150-404^ constructs, these fragments were PCR amplified and cloned into *BamH* I/*Kpn* I restriction sites of pQM-N-Tag A vector. Spastin-GFP construct was a kind gift from Dr. Elena I. Rugarli [7].

### Cell culture, immunofluorescence and immuno-precipitation experiments

The NIH-3T3 (ATCC Nr.: CRL-1658) and HeLa (ATCC Nr.: CCL-2) cells were cultured and maintained as previously described [12]. NSC34, a mouse embryonic spinal cord–neuroblastoma hybrid cell line with motor neuronal properties [47] was a kind gift from Dr J. Weishaupt (University of Goettingen, Germany). The cells were cultured and maintained in Dulbecco's modified Eagle's medium (DMEM) supplemented with 10% fetal bovine serum (FBS) and 1% penicillin/streptomycin/glutamine solution as previously reported [46]. The generation of mouse primary neuronal cells and culture conditions were described elsewhere [48]. For all the cell lines, transfection was done using Lipofectamine2000 reagent (Invitrogen) according to the manufacturer's instructions. Immunofluorescence and immunoprecipitation experiments were essentially performed as previously described [12], [46]. ImageJ program (http://rsbweb.nih.gov/ij/index.html) was used to measure the width of the cell body across the nucleus and the data were analyzed by Student's t-test application of GraphPad Prism4.0 (GraphPad Software).

### Sucrose density gradient experiments

For sucrose density gradient centrifugation, the NIH-3T3 cells overexpressing E2-ZFYVE27^WT^ were washed two times (10 min each) with PBS and incubated with HKME buffer (25 mM HEPES, pH 7.8, 150 mM potassium acetate, 2.5 mM magnesium acetate, 1 mM EDTA and protease inhibitor cocktail) containing 1% Big-CHAPS for 15 min. The cells were then harvested by scrapping them from plastic surface of the culture flask and incubated for 30 min on ice with mild vortex for cell lysis. The cell debris from the protein lysate was removed by centrifugation at 10,000 g for 10 min at 4°C and 200 µl of the resultant supernatant was applied on 5–30% sucrose gradients and centrifuged at 135,000 g for 4 hrs at 25°C on a Beckman coulter ultracentrifuge with TLS55 rotor. Approximately thirty gradient fractions of 50 µl (each) were collected from top to bottom and were analyzed by SDS-PAGE followed by immunoblotting with ZFYVE27 antibody (polyclonal antibody raised against the mouse ZFYVE27^213-344^ protein in rabbit as host). Molecular masses were calculated from the sedimentation of standard molecular weight markers: albumin, bovine serum (∼66 kDa), alcohol dehydrogenase, yeast (∼150 kDa) and apoferritin, horse spleen (∼450 kDa) purchased from Sigma-Aldrich.

### Subcellular fractionation and Triton X-114 membrane phase separation

Subcellular fractionation was performed with NSC34 cells. The trypsinized cells were suspended in a buffer containing 20 mM HEPES pH 7.8, 150 mM NaCl and protease inhibitor cocktail and were triturated by passing through 27-gauge syringe (15–20 times). The homogenate was centrifuged at 4°C for 5 min at 3,000 g to obtain post nuclear supernatant and the resultant supernatant was then centrifuged at 100,000 g for 60 min. The supernatant containing the cytosolic fraction (S-100) was separated and the pellet containing membrane fraction (P-100) was resuspended with the buffer to the original volume. Each subcellular fraction (20 µl) was then resolved in SDS-PAGE and immunoblotted with ZFYVE27 antibody.

The Triton X-114 phase separation was essentially performed as described previously [49]. Briefly, the membrane fraction (prepared as above) was mixed with pre-condensed Triton X-114 to a final concentration of 2% and incubated on ice for 30 min with occasional mixing. The detergent soluble fraction obtained after centrifugation at 16,000 g for 15 min at 4°C was subjected to aqueous and detergent phase separation at 37°C (cloudy point temperature) for 5 min and centrifuged at room temperature for 5 min at 16,000 g. The detergent phase containing integral proteins and aqueous phase containing peripheral proteins were separated and subjected to one more round of separation for enrichment. Finally, detergent and aqueous phases were pooled separately and the proteins were precipitated using standard acetone precipitation method [50]. After acetone precipitation, the protein pellets were resuspended and denatured in SDS-PAGE loading buffer and 20 µl of each fraction was analyzed by SDS-PAGE and immunoblotting.

### Liposomal Binding Assay

The liposomal binding assay was carried out by using pre-made PolyPIPosomes consisting of PtdIns3*P* moiety (Echelon Biosciences) as the substrate for ZFYVE27 protein. The full-length ZFYVE27 protein was produced by transient transfection of HeLa cells with c-Myc-ZFYVE27^WT^ construct. Also, a recombinant GST-ZFYVE27^300-404^ was produced in *E. coli* BL21 (DE3) strain and purified by glutathione sepharose-column (Sigma). The protein of interest (100 µg of HeLa cell extracts or 10 µg of purified protein) was diluted to 200 µl with binding buffer (50 mM Tris-HCl pH 7.6, 150 mM NaCl and 0.05% Nonidet P40), mixed with 10 µl of PolyPIPosomes and incubated overnight at 4°C. The next day, samples were centrifuged at 20,000 g for 1 hr at 4°C to precipitate the protein-liposome complex. The pellet containing protein-liposome complex was analyzed by Western blot with anti-ZFYVE27, EEA1 and GST antibodies.

## Supporting Information

Figure S1 **Validation of interaction of E2- ZFYVE27 (various domains) with c-Myc-ZFYVE27 by co-immunoprecipitation assay in mammalian cells.** The NIH-3T3 cells were co-transfected with full-length ZFYVE27 fused with c-Myc epitope tag and E2 tagged ZFYVE27 (full-length) or N-terminus (ΔC) or C-terminus (ΔN) or ZFYVE27 lacking the core interaction region (ΔHR3). The cell lysates were subjected to immunoprecipitation with c-Myc tag antibody and the resulting immunoprecipitants were analyzed in the immunoblot with E2 tag antibody (Co-IP: c-Myc; WB: E2). A portion of the cell lysates (input) was also subjected to immunoblot with E2 (WB: E2) tag antibodies to verify the protein expression of the indicated constructs. For mock experiments, the cell lysates were precipitated with non-specific IgG and subsequently analyzed by immunoblotting (as described above).
(TIF)

Figure S2 **Interaction of spastin with wild-type and truncated ZFYVE27.** The NIH-3T3 cells were co-transfected with GFP-Spastin together with either E2-ZFYVE27^WT^ or E2-ZFYVE27^ΔHR3^. Subsequently, the protein extracts were subjected to immunoprecipitation (IP) with E2-tag specific antibody or rabbit IgG (mock) and the resulting immunoprecipitants were analyzed by Western blot (WB) with GFP antibody. A portion of the cell lysates (input) was subjected to WB with both E2 and GFP antibodies to confirm the expression of indicated constructs.
(TIF)

Figure S3 **The mutant ZFYVE27^ΔHR3^ expression causes swelling of the cell soma.** Bar graph showing the average width of the cell body measured across the nucleus using ImageJ program in untransfected (control) or transfected with either ZFYVE27^WT^ or ZFYVE27^ΔHR3^ constructs in NIH-3T3 **(A)**, HeLa **(B)** and NSC34 **(C)** cells. Parenthesis indicates the number of cells (n) used for the analysis. Statistical significance is analyzed by Student's t-test (***P<0.0001).
(TIF)
