# Supplementary figures and images for: Oligomerization of ZFYVE27 (Protrudin) Is Necessary to Promote Neurite Extension
Source: PLoS One. 2011 Dec 28;6(12):e29584. doi: 10.1371/journal.pone.0029584 (PMC3247280; doi:10.1371/journal.pone.0029584)

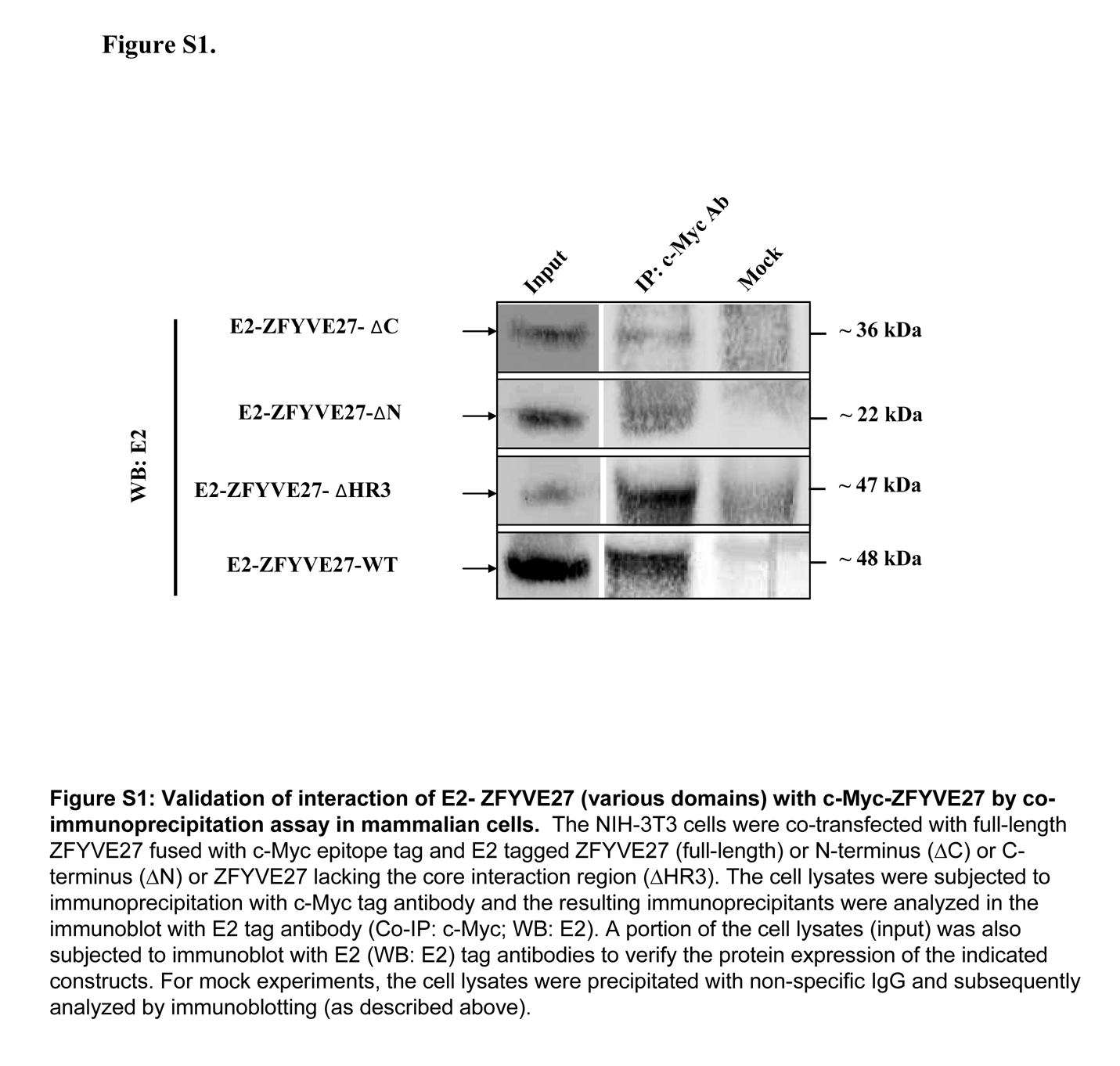

Supplement: Figure S1 — Validation of interaction of E2- ZFYVE27 (various domains) with c-Myc-ZFYVE27 by co-immunoprecipitation assay in mammalian cells. The NIH-3T3 cells were co-transfected with full-length ZFYVE27 fused with c-Myc epitope tag and E2 tagged ZFYVE27 (full-length) or N-terminus (ΔC) or C-terminus (ΔN) or ZFYVE27 lacking the core interaction region (ΔHR3). The cell lysates were subjected to immunoprecipitation with c-Myc tag antibody and the resulting immunoprecipitants were analyzed in the immunoblot with E2 tag antibody (Co-IP: c-Myc; WB: E2). A portion of the cell lysates (input) was also subjected to immunoblot with E2 (WB: E2) tag antibodies to verify the protein expression of the indicated constructs. For mock experiments, the cell lysates were precipitated with non-specific IgG and subsequently analyzed by immunoblotting (as described above). (TIF) [file pone.0029584.s001.tif]

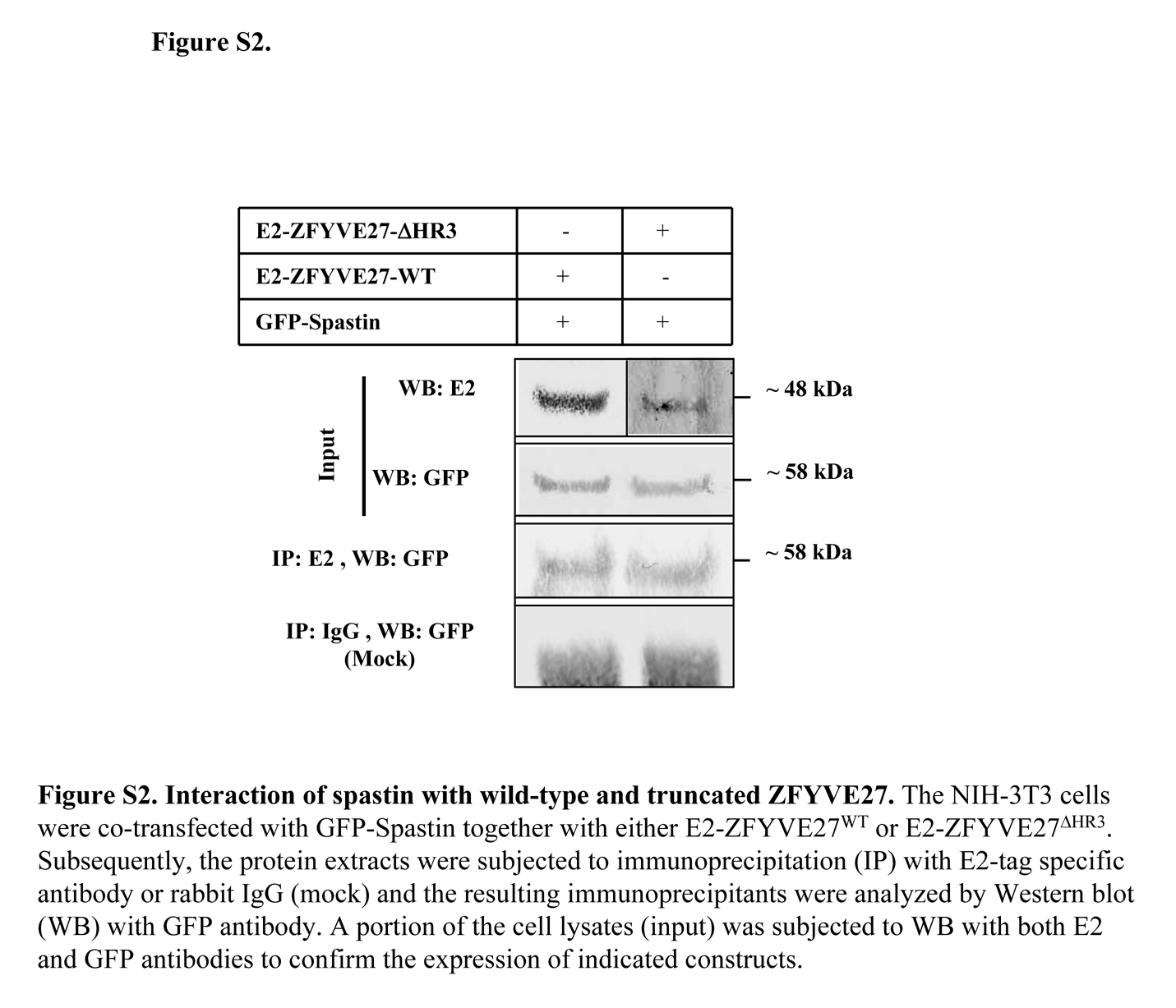

Supplement: Figure S2 — Interaction of spastin with wild-type and truncated ZFYVE27. The NIH-3T3 cells were co-transfected with GFP-Spastin together with either E2-ZFYVE27WT or E2-ZFYVE27ΔHR3. Subsequently, the protein extracts were subjected to immunoprecipitation (IP) with E2-tag specific antibody or rabbit IgG (mock) and the resulting immunoprecipitants were analyzed by Western blot (WB) with GFP antibody. A portion of the cell lysates (input) was subjected to WB with both E2 and GFP antibodies to confirm the expression of indicated constructs. (TIF) [file pone.0029584.s002.tif]

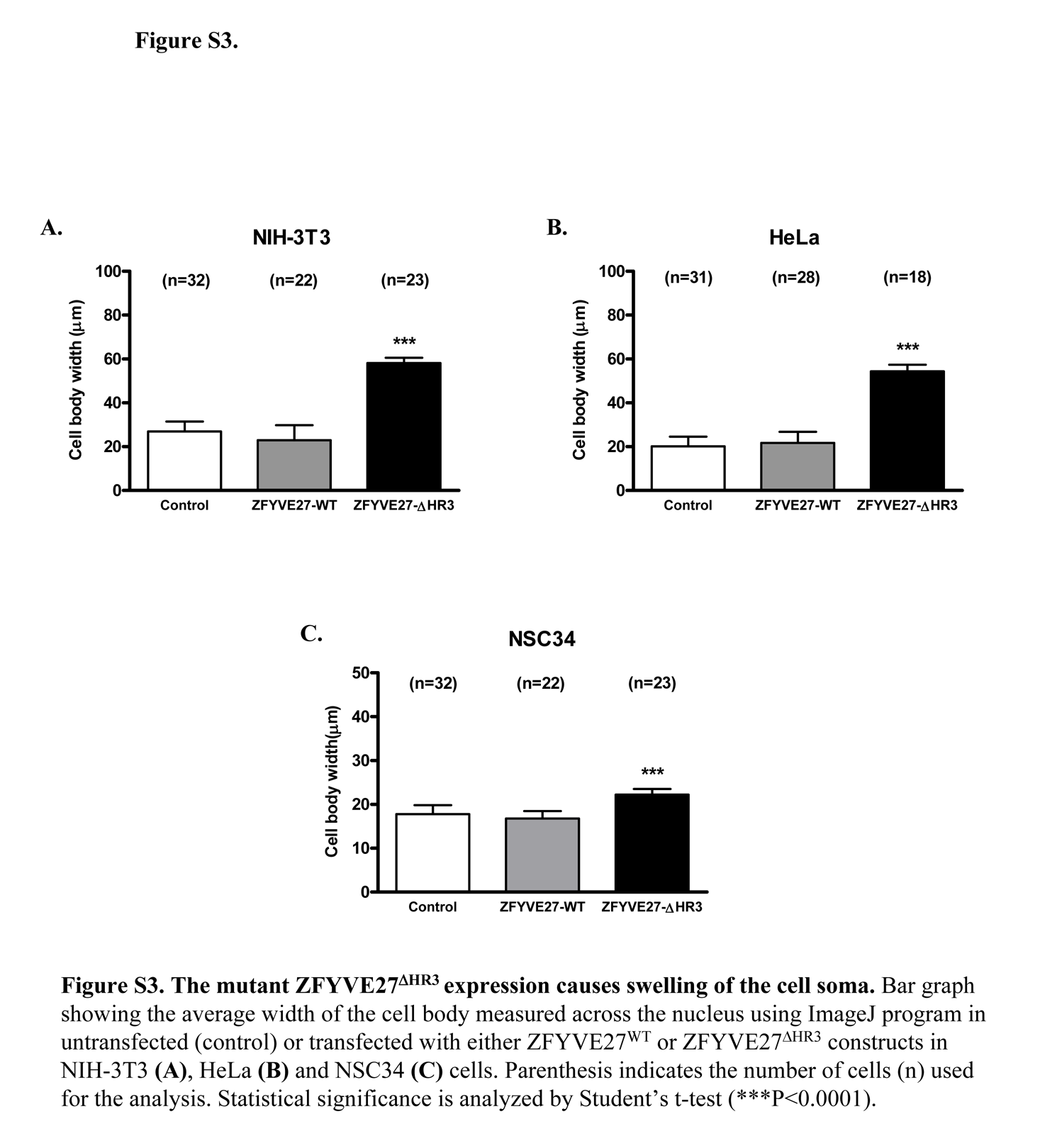

Supplement: Figure S3 — The mutant ZFYVE27ΔHR3 expression causes swelling of the cell soma. Bar graph showing the average width of the cell body measured across the nucleus using ImageJ program in untransfected (control) or transfected with either ZFYVE27WT or ZFYVE27ΔHR3 constructs in NIH-3T3 (A), HeLa (B) and NSC34 (C) cells. Parenthesis indicates the number of cells (n) used for the analysis. Statistical significance is analyzed by Student's t-test (***P<0.0001). (TIF) [file pone.0029584.s003.tif]
